# The poke test in lower limb fasciotomy: a potentially limb saving technical note

**DOI:** 10.1308/003588412X13373405387050d

**Published:** 2012-10

**Authors:** I Pallister, S Rahman, S Atherton

**Affiliations:** Abertawe Bro Morgannwg University Health Board,UK

## BACKGROUND

Delayed or incomplete fasciotomy may result in limb loss or even death in severe polytrauma.^1^ The angiosomal blood supply of the anterior compartment renders it particularly vulnerable if not correctly decompressed.^2^ Swelling and local trauma distort anatomy significantly, making it possible to ‘miss’ the intended compartment without some simple means of double-checking. The anterior and lateral compartments may be decompressed via separate fascial incisions through an axial skin incision (running midway between the fibula head and the tibial tuberosity proximally, and the lateral malleolus and the anterior tibia distally).^2^ Alternatively, the anterior compartment is decompressed via an incision 2cm lateral to the tibial crest. The lateral compartment is then decompressed into the anterior by incising the intermuscular septum, extremely difficult in the presence of severe swelling.^3^

## TECHNIQUE

**Figure 1:**
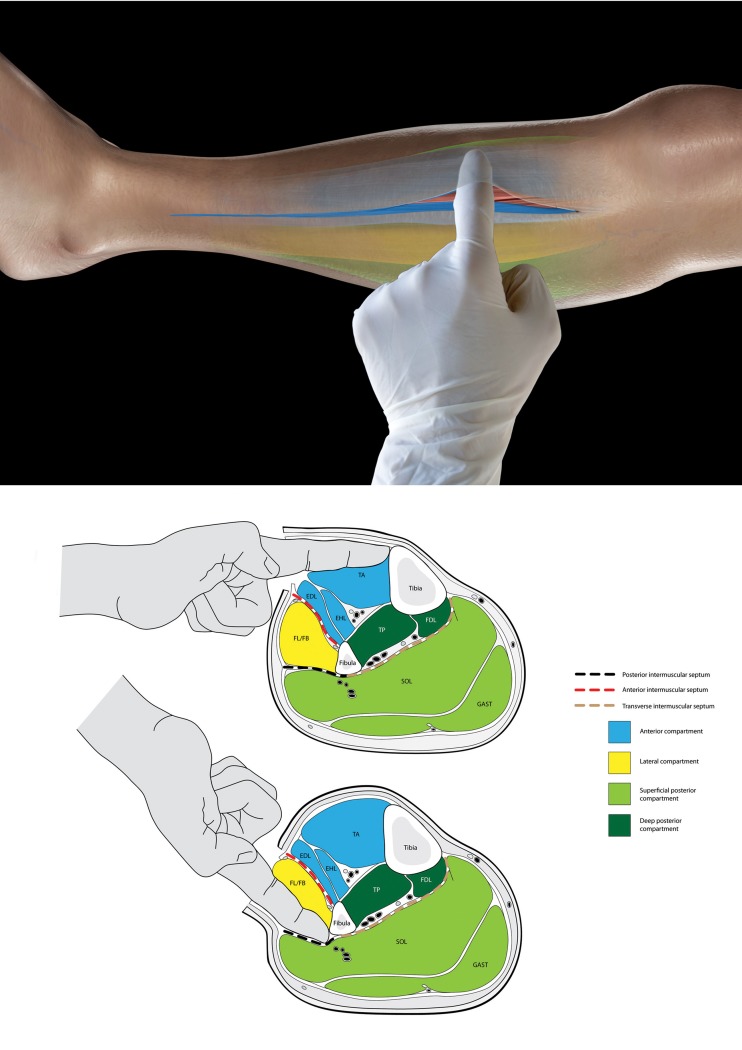
Top diagram: The surgeon’s finger has been introduced through an incision in the fascia and poked across to touch the tibia, confirming this is the anterior compartment. Bottom diagram: The finger is again introduced through a second fascial incision and palpates the fibula, confirming entry into the peroneal compartment. If the surgeon entered the peroneal compartment (believing it to be the anterior due to swelling and distortion) and attempted to poke the tibia, this would prove impossible because of the intermuscular septum. Correspondingly, the septum prevents palpation of the fibula from the anterior compartment.

After entering the deep fascia, a finger is inserted and advanced towards the midline, superficial to the muscle but deep to the fascia. If the finger is in the anterior compartment, it will touch the tibia easily. If, however, the finger is in the lateral compartment, this will be impossible. Furthermore, if the direction of the finger is reversed and advanced, the fibula will be felt (Fig 1). It is imperative to ‘poke’ the finger in and never to sweep it along the length of the wound. This would avulse perforators with potentially disastrous results.

## DISCUSSION

By using this simple poke test, the surgeon can identify swiftly and with certainty which compartment he or she has entered and decompressed.
